# Lay health advisers: scoping the role and intervention landscape

**DOI:** 10.2147/JHL.S134464

**Published:** 2017-07-28

**Authors:** Susan M Carr, Monique Lhussier, Natalie Forster

**Affiliations:** 1 Public Health Research, Department of Social Work, Education, and Community Wellbeing, Northumbria University, Newcastle upon Tyne, UK; 2 Department of Education and Training, Federation University Australia, Ballarat, VIC, Australia; 3 Fuse, Centre for Translational Research in Public Health, Newcastle upon Tyne, UK

**Keywords:** realist logic, lay health advisers, inequalities, hard-to-reach populations, service design

## Abstract

The use of lay health advisers has become an established approach within public health, in particular for impact on health inequalities and engaging socially excluded groups. Evidence on how differences in terms of the multiple role dimensions impact the outcomes of programs is limited. This creates ambiguity for decision makers on which roles should be implemented in different contexts for different needs. This paper applies realist logic to an inquiry to explore the mechanisms that may operate in lay-led intervention models and understand how, why, and in what respect these lead to particular outcomes. It draws on a project focusing on health-related lifestyle advisers and further insights gained from a subsequent related project about outreach with traveler communities. Analysis highlights multiple and potentially interacting aspects of lay health-adviser roles that may influence their success, including characteristics of lay health advisers, characteristics of target populations, purpose or intent of interventions, and how advice is given. A model is proposed from which to examine the contexts and mechanisms of lay health advisers that may impact outcomes, and is subsequently applied to two examples of reported lay health-adviser interventions. The combination of skills and characteristics of lay health advisers must be considered when planning which interventions might be appropriate when targeting specific needs or target populations. Focus only on the peer/layperson distinction may overlook other potentially important skills and mechanisms of action integral to lay health-adviser roles.

## Introduction

Lay health advisers (LHAs) are seen as an intervention with significant potential to challenge stubborn health inequalities in the health- and community-service sectors.^1^–^5^ The value of such roles is assumed to lie in the qualitatively different relationships through which any intervention is delivered. Evaluations have provided evidence of impact,^6^,^7^ although pathways to impact require further articulation.^8^ This leaves leaders and managers with inadequate information on which to make decisions about the potential of LHAs in addressing health and social needs. The specific contribution of this paper is to draw on realist principles to interrogate such roles to explain more about how and why they might lead to favorable outcomes. We map out a range of features, such as employment status, focus of intervention, and LHA specification, and highlight potential consequences of selecting different configurations. We anticipate that such detail will benefit the development and evaluation of interventions to match roles better to specific needs and targeted outcomes, thereby maximizing attempts to reduce inequalities.

We draw principally on a project focusing on health-related lifestyle advisers and further insights gained from a subsequent related project about outreach with traveler communities.^9^,^10^ We are adopting an inclusive approach to role titles, reflecting the multiple roles and models emerging in practice operating in diverse populations and settings. Overarching criteria for inclusion are public health-focused activities delivered by a layperson or peer to a population who are considered hard to reach through standard service provision.

Acknowledgment that current service provision does not address all health needs has led to increasing attention on layperson-led approaches. This is also fueled by workforce issues, levels of service demand,^11^,^12^ and persisting health inequalities.^11^–^13^ One of the most dominant debates to date relates to exposing what distinguishes these types of roles from other, professionally led approaches.^5^ There is not a single accepted definition, but it is possible to identify three commonly accepted purposes of the role: access to communities or individuals, access of marginalized communities into health and social care systems, and alternative delivery mechanisms to professional providers.^9^ A core quality would thus seem to be ownership of some type of indigenous knowledge that could relate to behavior, community, or culture.^14^

Moving on from trying to define and distinguish the LHA role per se, there is also a need to put the microscope on differences and distinguishing characteristics among LHA roles. Treating these roles as having a single distinguishing denominator, the lay or peer characteristic, may be inadequate, and multifactorial descriptors may be more useful. It is well accepted that the design, delivery, and evaluation of public health interventions are complex, in that they are implemented in dynamic and shifting contexts and are often locally determined in order to respond to the needs of particular populations.^15^ Decision makers are often “… obliged to take action to tackle health inequalities, but with a totally inadequate evidence base to inform their decision making — leading them to invest in both evaluated and unevaluated interventions”.^16^ In addition, the urgency to act to address health inequalities has often resulted in insufficient attention to the design and introduction of policies. Realist approaches, which offer information on four key questions – By whom is health advice given? To whom is health advice given? For what purpose is health advice given? How is health advice given? – assist decision makers to apply more “bespoke” strategies and funding and are thus welcomed in the fight to address inequalities effectively.

## Materials and methods

The application of realist logic highlights the processes of layperson-led intervention models in order to understand how, why, and in what respect these lead to particular outcomes.^17^ This includes an explicit acknowledgment of the impact of the political, organizational, historical, social, and economic contexts within which interventions are developed and so to increase the potential to respond to local health-inequality challenges with greater levels of specificity.^18^

Layperson health advice has so far been conceptualized as various combinations of process resources, such as communication aids and training. Our analysis considers which of these resources are relevant and necessary to produce desired outcomes, how they interact with each other, and how they might be optimally implemented. Such considerations reveal information about context, mechanisms, and desired outcomes. Interaction developed between LHAs and their target group are at the core of the intervention. The interpersonal relationships that are created are assumed to be fundamental to achieving success, and thus feature highly in our considerations.

The detail of the scope and inclusion criteria of the primary data on which we have drawn is detailed in reports.^9^,^10^ In summary, a search of electronic databases was made (ASSIA [Applied Social Sciences Index and Abstracts], Embase, NHS EED, Medline, and PsycInfo), as well as searching journals and reference lists. Identified studies were scanned by two reviewers, their quality assessed, and then selected for inclusion in the reviews. Data were abstracted from each study, guided by an agreed protocol.

Drawing on realist methodology,^17^ we further developed our synthesis activity from these studies by posing four questions of the data:

- By whom is health advice given?
- To whom is health advice given?
- For what purpose is health advice given?
- How is health advice given?

The findings are summarized under these headings and tabulated to highlight the potential consequences for LHA interventions, highlighting some of the implicit tensions and conflicts. We were thus able to expose the continuum of role models and then to proffer and challenge some suggestions of mechanisms of action. From these questions, a model is proposed in the discussion and illustrated by two examples found in the literature.

## Results

### By whom?

In Table 1, we identify two key facets of the LHA role: pragmatic employment features and person-specific features. In terms of pragmatics, we include level of qualification, time commitment, and remuneration issues. With respect to person specifics, we distinguish by “layness”, shared experiences, or a shared community. We explore the consequences of these different dimensions for the roles, and highlight where ambiguity may exist and where further clarity may bring benefit.

### To whom?

Two key distinguishing features with respect to whom the LHA delivers a service may be made. That is whether the approach is population-wide or targeted at a specific group or community. When the LHA is providing a universal service across a population, the degree of homogeneity will influence how many of the characteristics detailed in Table 1 may be active. A review of the literature has allowed us to identify seven dimensions of targeting:

1. people with shared belief, cultural background, ethnicity
2. people living in a restricted geographical area
3. people with a shared illness experience or disability
4. people at a similar stage in life
5. people engaging in risky behaviors
6. people seeking to engage with services
7. people living in specific/complex circumstances, eg, homeless people, prisoners, those living in refuges

It may be, of course, that an individual LHA may actually be drawing on multiple-dimension combinations and that this is often considered to be the “ideal” in terms of having the maximum level of “peerness”. The complexity of attempting to identify the active mechanism in the LHA role is thus further exposed.

While the literature clearly describes distinct population groups, we would suggest that distinguishing categories of target groups is somewhat a red herring, in that the mechanism that is most likely to lead to the successful delivery of a message is likely to be influenced by the interplay between the individual LHA and the targeted group, rather than in their separate characteristics. This interplay needs to be mapped out in light of the overall purpose of the intervention.

### Purpose or intent of LHA interventions

We suggest that purpose and intent may be discussed at two levels (Table 2). One level is the stated purpose, eg, to increase screening uptake or deliver specific lifestyle advice. A second superimposed aim or level is the purpose of the LHA as a specific intervention strategy or approach, eg, to access a hard-to-reach population, to provide expert patient input, or to increase social capital.

### How is advice given?

In order to attempt to expose the “how” of layperson health advice, we distinguished by focus (individual, interpersonal, and community levels of action) and process (information giving, nurturing, or supporting) (Table 3).

## Discussion

A wide diversity of LHA roles exist, fashioned on single- or multiple-intervention models and theoretical frameworks. We have mapped the complexity and highlighted some of the implicit tensions and conflicts. Our findings suggest that a unilateral focus on the peer/layperson distinction may actually cloak some potentially important skills and mechanisms of action integral to LHA roles. This leads us to suggest that a useful development in the field of layperson-led interventions is to consider the combinations of skills and characteristics that make up LHA roles. This would provide a much better evaluation landscape by moving away from a very generic mind-set about LHAs, which can encourage neglect of some of the often-hidden multiple-role dimensions. It would assist in developing thinking about how the multiple elements of LHA roles may be packaged to respond most effectively to specific needs.

The theoretical underpinnings of LHAs may be categorized into three themes: individual behavior change, social learning or influence, and communication or learning approaches. Crucially, most of the studies included in the health-related lifestyle-adviser role review had a theoretical underpinning that exploited links and influences.^9^ The importance of such theories not only presents us with commonsense appeal but has also been confirmed in a review of outreach interventions in traveler communities.^10^

The four questions we posed can be mapped onto the context + mechanism = outcomes algorithm used in realist approaches.^17^ This algorithm is an “heuristic used to generate causative explanations pertaining to outcomes in the observed data.”^19^ Interventions are theorized to work through altering the context in which they are implemented to generate underpinning mechanisms that lead to observable outcomes. Following this approach, “to whom” can inform understanding of the contexts within which interventions occur. “How” and “by whom” can help catalogue the resources deployed in LHA interventions, and a “what for” question can help expose intended outcomes. From there, a realist logic of analysis can be applied, which links these constructs in explanatory statements.

Therefore, we propose that applying realist analysis logic offers a useful approach to managing the breadth of data required to commission, develop, or evaluate LHA roles. This is not offered as a clean-cut formula to identify the most appropriate LHA role to meet a particular need. Rather, it should be emphasized that it requires prioritization decision making and trade-offs between intervention dimensions. It does, however, move some way to ensuring that all the potential mechanisms of action are recognized and so have potential opportunity for effectiveness.

The application of this logic is demonstrated by working through two exemplars of LHA interventions reported in the literature. This paper does not seek to scrutinize all possible permutations of the characteristics of LHA interventions and patterns suggesting favorable combinations of the different components. Rather, the exemplars are illustrative of how the framework may be applied, in order to consider more explicitly the necessary components when building and evaluating LHA interventions. Examples of reported interventions are drawn from the studies included in previous analyses, and were selected due to their contrasting positions along the continuums of intervention-delivery mode (low formality vs high formality), degree of targeting (one to one vs general population), and differences in the type of layperson/peer worker utilized (shared risk behaviors vs shared sex and ethnicity).^20^,^21^ Both focus on HIV prevention, one through safer practices in injecting drug users in the US, and the other through safer sexual behavior in a group of Roma men in Bulgaria. The geographical, cultural, and ethnic contexts are thus widely different, but both studies explicitly build on the strengths of social networks, enabling a detailed analysis of the explanatory potential of such a promising mechanism of action.

### Exemplar 1: Evaluation of a volunteer AIDS outreach intervention – aids and behavior

This study was a qualitative evaluation of a network-oriented outreach program for HIV prevention delivered by African-American drug users in Baltimore, US, trained to promote HIV prevention in their neighborhoods.^20^ It reflects social and contextual factors influencing outreach encounters in this setting, in particular between older drug-using LHAs and adolescents.

#### By whom

LHA workers underwent intensive training consisting of ten sessions, each lasting 90 minutes, on sexual- and drug-risk reduction and a social identity component. Remuneration was provided for participation in the training, interviews, and for the time spent with ethnographers during partnered visits. LHAs delivered interventions opportunistically, and were not employed. LHAs were part of the networks within which they were promoting HIV prevention, and the majority had personal experience of using illegal drugs.

#### To whom

The target population shared cultural backgrounds and engagement in risky behaviors, and had similar socioeconomic status. However, these descriptors mask financial, sex, and age divisions within the community, which impacted how well LHAs were able to deliver messages and how well they were received.

#### For what purpose

The purpose of the intervention was to promote hygienic needle use among injection-drug users, in order to prevent HIV.

#### How

**Figure 1:**
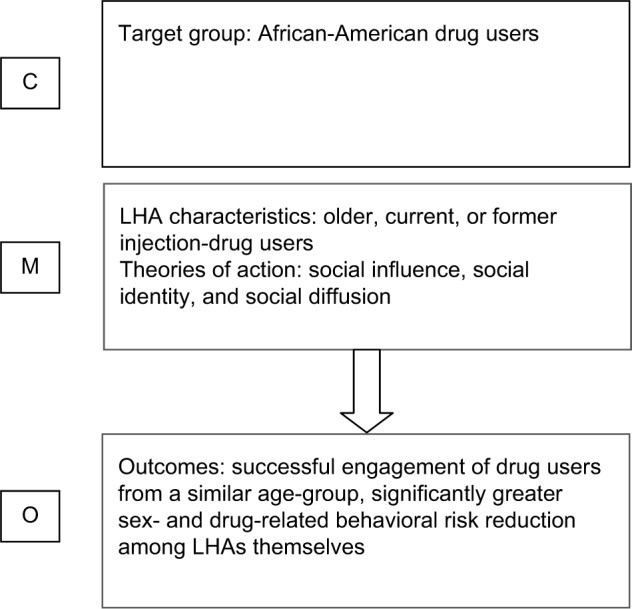
Context–mechanism–outcome (C-M-O) logic model applied to Dickson-Gómez et al.^20^ **Abbreviation:** LHA, lay health adviser.

LHAs delivered interventions to individuals or small groups on an opportunistic and informal basis, in places and times appropriate to the outreach worker. The intervention focused on the provision of targeted information on HIV prevention and kits of preventive equipment. However, drawing on theories of social influence, social identity, and social diffusion, the study sought to capitalize on the sense of community identity among African-American drug users and increase participants’ sense that they could positively influence the health and well-being of their family and social networks. A summary of the context–mechanism–outcome logic as applied to Dickson-Gómez et al is presented in Figure 1.^20^

LHAs were selected on the basis that they were peers with both a shared community and risky behavior experience. However, while LHAs reported successful outreach encounters with those who were in a similar age-group, they found it more difficult to relate to adolescents. Older male injection-drug users were stigmatized as a result of their relative position of greater impoverishment, in contrast with non-drug-using adolescents who were trading drugs and had greater financial status. As a result, these older drug users lacked credibility, as their drug use was perceived as a sign of failure, and attempts to offer outreach were seen as challenging the hard-earned street-level respect of younger community members. Outreach workers who were mature women and who adopted the role of a surrogate mother were more accepted by adolescents. This highlights the fact that an assumption of cultural homogeneity based on ethnicity, geography, or engagement in risky behavior only is insufficient to lead to successful outcomes.

Prior understanding of the types of social bonds linking people within a target group is key in identifying promising levers for change through social influence. Indeed, older male current or former drug users proved successful conduits for message delivery in people from their own age-group, but not adolescents, who for the main part were not using, but rather selling drugs. Older women had some success with adolescents, because they were perceived as caring mother figures. Concomitant with sex and age, communication styles were an important factor, as directive and moralistic approaches alienated adolescents, but a more caring perspective was respected and listened to.

### Exemplar 2: Prevention of HIV and sexually transmitted diseases in high-risk social networks of young Roma (Gypsy) men in Bulgaria – randomized controlled trial

This research was conducted in one of the largest Roma populations in Bulgaria.^22^ It included an initial ethnographic phase, designed to map out significant networks within the traveler community.

#### By whom

Observations were undertaken by people with whom the population were very familiar, so that social circles and the person at their social and affective core could be identified. The term “index” was applied to these people, and they were then the means of accessing and recruiting social networks. The research team was provided with a network list by each index. Using such criteria as trust and spending time together, researchers were then required to identify their least and most preferred individuals. Network leaders were then distinguished using a sociometric analysis. These leaders then received education and guidance on approaches to counseling and advising other members of the network on reducing HIV-risk behavior. Social networks included between three and nine men. Two-hour training sessions were initially provided for a period of 5 weeks and then reduced to every 2 weeks and then every 2 months. Indices were incentivized for taking part, but received no other formal payment.

#### To whom

The Roma community are both marginalized and disadvantaged, and are the largest ethnic-minority population in Eastern Europe. They also experience significant deprivation living in settlements that are separate from the majority community.^5^ These people have a life expectancy 10–15 years shorter than the neighboring population. Vulnerability to HIV/AIDS and other sexually transmitted diseases is an issue of increasing concern. This health need is unlikely to have been identified by the Roma population, given the taboo nature of their attitudes to sexual behaviour.^23^,^24^

#### For what purpose

The population are encouraged and facilitated to change their behavior through group leaders exploiting opportunities to exert social influence. A central tenet of this approach is the notion that Roma populations held significant distrust of outsiders: “… advice and recommendations coming from personally known network members … carry credibility and influence.” Adjustments in social norms were identified as the reasons for the greater robustness of outcomes reported at 12 months when compared with those at 3 months. Behavior changes related by the social network were supported by a reduction in the incidence of gonorrhea. Control-group comparisons showed a significantly greater reduction in the prevalence of reported unprotected intercourse (*P*=0.01) and significantly increased knowledge of the risk of AIDS, positive attitudes to condoms, and strength of intentions to reduce risk behavior in the intervention group The group experiencing the highest levels of effectiveness were reportedly those at highest risk through casual partner relationships.

#### How

**Figure 2:**
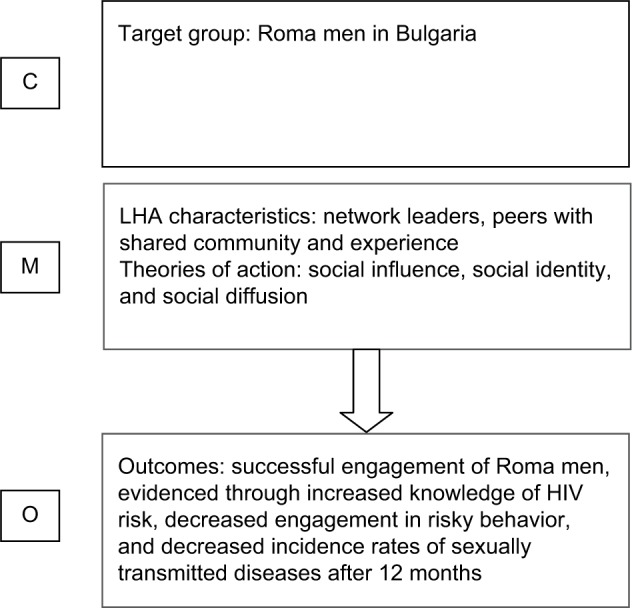
Context–mechanism–outcome (C-M-O) logic model applied to Kelly et al.^22^ **Abbreviation:** LHA, lay health adviser.

The intervention relied heavily on strong social ties and high levels of trust. However, the community was afforded the option of sharpening or determining the focus of the intervention. This might have provided an opportunity to ensure that the provision fitted well with perceived need. Leaders had an average of 6.8 opportunistic conversations with any given network members (corroborated by the members themselves). The leader was known and trusted by the group. Factors that may have made the intervention have relevance for them could have been their higher levels of risky behavior activity and pressure to conform to the evolving social norm. The chain of action responsible for the effectiveness of the intervention is conceivably group-level social influences facilitating engagement from individual risk takers and reported behavior changes. A summary of the context–mechanism–outcome logic as applied to Kelly et al^22^ is presented in Figure 2.

## Conclusion

The provider and recipient populations for layperson health advice are complex. Realist logic analysis has highlighted the need to move away from largely unidimensional descriptors of either LHAs or their target groups to understand the premise of their relationship and how levers of change may be activated. Such degrees of specificity with respect to role and need allow the intent to be more clearly articulated, and consequently the underlying mechanism of actions that are likely to lead to effective intervention to be understood. Crucially, the dynamic nature of the relationship should be recognized, so that characteristics can be selected to provide tailored responses to a range of stubborn inequalities.

The model presented in this paper acknowledges the continuing challenge of defining a community from the outside, and thus highlights the necessity of considering both relationships with the target group and commonality of experiences. Affiliation with a host organization, employment, or remuneration all impact on the intervention and the extent to which a true alternative to standard-service approaches is facilitated.

## Figures and Tables

**Table 1:** By whom: characteristics of LHAs

Employment features	Consequences and questions
Training	Enhanced training can create a range of consequences, in addition to skill-set development. It may enrich the potential of the LHA to undertake a range of tasks, but it may also challenge their degree of “layness” or increase social distance between them and other community members, and so impact the very characteristics initially brought to the role.
Organization in which LHA is based or affiliated	The choice of host organization is a significant factor in facilitating role “fit”, as it may steer the role toward an established model, rather than allowing more “bespoke” development to fit population needs. This could be significant if the target population have a history of limited service participation.
Role boundaries	Movement in and out of role may be facilitated when there is a discrete target audience and the opportunity to deliver advice may be presented at defined times, eg, an annual festival. When the focus is less targeted and more opportunistic, then “in role/out of role” clarity may be more difficult to achieve. Movement in and out of role may not be a desired outcome, and the ideal may be that the person actually integrates LHA aspects into their everyday role.
Remuneration	A variety of approaches to remuneration exist, from out-of-pocket expenses, to a “salary” for a service that could alternatively be provided by an employee of the service. There are clearly issues to consider about how remuneration functions in differentiating these roles and impacting how the LHA perceives their role and how others perceive them.

**Person-specific features**	**Consequences and questions**

Peer with common personal experience and drawing on scientific knowledge	The Expert Patients Programme in England is a self-management program for people who are living with a chronic (long-term) condition, run by tutors with the condition. Shared experience is assumed, and in some cases has been demonstrated to facilitate communication and impact on the recipient. This is accompanied by intensive training so that self-management strategies communicated are in line with evidence recommendations.
Peer with common personal experience drawing on experiential knowledge	Advice is experience-based, and there is thus a variety of personal points of connection, eg, the experience may be being female, a female who is a mother, a female who is a traveler community member, a female with diabetes, or multiples of these. Drawing on experiential rather than scientific evidence does not sit easily in an evidence-based environment, and this is an example of the potential for paradigm conflicts inherent in LHA roles that are aligned with formal, established delivery systems.
Peer with shared community	“Community” can be defined on a number of dimensions, such as geography, culture, faith, and sex. Some level of specificity is thus required to clarify which community or communities are shared, and they may of course be multiple. Some shared community experiences could be episodic, eg, attending a weekly faith service. Others could be continual, such as living in a specific geographic area or working in the same organization.
Peer with both shared community and experience	Increasing levels of specificity are present in these roles, such as female smoker living in a particular geographical area. Such detail may be helpful in understanding the mechanisms of action involved in the LHA process.
Not peer, but layperson	The distinguishing characteristic here is not shared experience, but rather not being a professional. Not being a professional may relate to professional training, as well as the social and economic divisions that may exist between the service provider and recipient populations. It is important to highlight that this dimension may actually be the active variable in the other roles listed here, but could in essence be masked by the assumption that it is “peerness” rather than “layness” that is the defining characteristic.

**Abbreviation:** LHAs, lay health advisers.

**Table 2:** Purpose and intent of LHA interventions

Health outcome	Consequences and questions
Targeted and focused health topic or behavior	A distinction should be made between behaviors that require a one-off attendance at an event (eg, screening or vaccination) and those that require ongoing engagement and longer-term commitment (eg, diet, smoking, and physical activity), as different LHA characteristics may be required.
Generic well-being and health promotion	This is clearly a difficult outcome to measure and thus gauge impact. It may be that the LHA may facilitate movement of health and health-related behaviors on to an individual’s agenda, and thus make the population more receptive to other forms of intervention. Indeed, some providers have interpreted the LHA role as one of “priming” often-disengaged populations to be more receptive to participating in more standard or mainstream service provision.
Increasing social capital in a population	This requires the highest degree of engagement behavior from the targeted community.

**LHA intent**	**Consequences and questions**

Sharing personal expertise supported by scientific or experiential knowledge	Examples could include the Expert Patients Programme, where improved self-management of diabetes is the intended outcome, or a breast-feeding support group, where duration of breast-feeding is the intended outcome.
Gaining access to hard-to-reach populations	It may be that the LHA is in such a privileged position that they are the only access point to some populations, eg, sex workers, homeless people, nonattendees. In these cases, gaining access and engaging with the group is an outcome in itself, which may in the longer term lead to behavior change or improved well-being.

**Abbreviation:** LHA, lay health adviser.

**Table 3:** Focus and process of LHA interventions

Focus	Consequences and questions
Individual	The opportunity to offer “bespoke” advice may be increased when delivered at the one-to-one level. Opportunistic interventions may also be more possible, so advice can be delivered when the LHA assesses a receptive opportunity or when it is specifically sought from the LHA by the recipient.
Group	Group communication may be opportunistic, but it is more likely to be in the form of an arranged encounter, and so may not necessarily have the luxury of attuning to receptiveness. A group approach may engage the LHA in addressing peer or wider community influences on health-decision making, and so could be seen to expand the complexity of the intervention. An additional level of peer education and support often occurs among group members, further enhancing complexity, but potentially offering additional benefits.
Community	The LHA could be working to improve and facilitate health-enhancing choices in the wider environment and so be active with agencies and organizations, as well as individuals or groups. This may require considerable political awareness, community and interagency perspectives, and negotiation skills.

**Process**	**Consequences and questions**

Targeted information giving	This is likely to require that the LHA has a definitive knowledge base on a specific subject. It could also include responsive signposting to specialist providers.
Generic information giving	This is likely to require that the LHA has a diverse knowledge base on multiple subjects and be able to assess and prioritize knowledge or information needs.
Nurturing or supporting	The LHA may draw on established or establishing network structures, rather than functioning at an individual agency level.
One-off contact	This is likely to demand high impact in a short period or signposting and dissemination of health-promotion materials, eg, leaflets.
Iterative contact	This demands relationship development and termination skills and the ability to assess the appropriate velocity of the change process.

**Abbreviation:** LHA, lay health adviser.
